# Strengthening Critical Health Infrastructure—One Road to Climate Resilience

**DOI:** 10.1002/puh2.70177

**Published:** 2025-12-11

**Authors:** Ana Raquel Nunes

**Affiliations:** ^1^ Warwick Medical School University of Warwick Coventry UK

**Keywords:** climate change, extreme weather events, healthcare infrastructure, public health adaptation, sustainable health systems

## Abstract

**Objectives:**

This short communication examines structural and systemic vulnerabilities in healthcare infrastructure in the context of climate‐related hazards and considers strategies for strengthening health infrastructure resilience, contributing to the long‐term sustainability of public health systems.

**Study Design:**

A descriptive analytical approach.

**Methods:**

Insights are drawn from (1) literature on climate resilience in healthcare infrastructure and (2) international frameworks addressing climate change adaptation in health systems until February 2025.

**Results:**

Healthcare infrastructure is increasingly vulnerable to extreme weather events, energy disruptions and cascading failures. Current infrastructure planning is often fragmented and reactive, prioritises short‐term concerns, lacking alignment with long‐term resilience goals. Areas that can support resilience include climate‐adaptive healthcare designs, sustainable energy integration, cross‐sectoral collaborations and enhanced policy frameworks. Several actions that can contribute to strengthening resilience, including potential strategies for implementation, are outlined. Coordinated efforts across health, urban planning and environmental sectors will be essential.

**Conclusions:**

As climate‐related hazards intensify, strengthening healthcare system resilience will require proactive planning, integrated policies and sustainable infrastructure investments. Without further action, the capacity of healthcare facilities to provide essential services during disasters could be significantly compromised.

## 1 Introduction

Climate change poses significant threats to healthcare infrastructure globally, with increasing frequency and intensity of extreme weather events, such as floods, heatwaves and storms, which disrupt service delivery [1, 2]. These threats compromise health systems’ ability to deliver essential services. Disruptions to health infrastructure, such as power outages, flooding of critical services, loss of access or facility damage, have been associated with increased morbidity and mortality during extreme events, particularly among vulnerable populations [1, 2]. Addressing these vulnerabilities will require a shift from reactive responses to proactive strategies that integrate climate resilience and sustainability principles into healthcare systems [2].

Healthcare systems are increasingly susceptible to disruptions from extreme weather events, rising temperatures and cascading failures across interconnected systems [1, 2, 3, 4, 5]. These disruptions have the potential to strain resources and negatively impact health outcomes, especially for vulnerable populations [1]. To help ensure continued service delivery, integrating climate resilience into infrastructure planning is imperative.

Despite growing calls for climate‐resilient health systems, most infrastructure planning remains short‐term, siloed and insufficiently climate‐informed. This short communication explores strategies targeting health infrastructure, highlighting their role in supporting public health continuity amidst accelerating climate threats. Specifically, it addresses the conceptual gap concerning limited operationalisation of resilience within healthcare infrastructure design, highlighting that existing adaptation frameworks inadequately integrate systemic flexibility into infrastructural planning.

## 2 The Need for Climate‐Resilient Health Infrastructure

Strengthening the resilience of critical infrastructure is essential for maintaining public health in the face of growing environmental challenges. Climate change, extreme weather events and public health emergencies increasingly place healthcare systems worldwide under pressure, highlighting the importance for integrated and adaptive planning [6, 7]. Resilient healthcare infrastructure must be able to withstand, adapt to and recover from disruptions while maintaining essential services. Although adaptation often refers to infrastructural and technological adjustments, resilience encompasses the broader capacity of health systems to anticipate, absorb and transform in response to climatic shocks [8]. This short communication therefore advances a vision of resilience as systemic flexibility, complementing adaptation's structural orientation.

## 3 Pressures From Climate Extremes on Healthcare Systems

Healthcare systems are facing increasing pressures due to extreme weather events, rising temperatures and energy insecurity [3, 4]. Integrating climate resilience measures—such as energy‐efficient cooling, flood‐resistant designs and redundancy in critical systems—will help to maintain service continuity. However, infrastructure policies often focus on immediate operational concerns rather than long‐term climate adaptation [5]. Taking a forward‐looking approach that incorporates climate projections, risk assessments and sustainability objectives will assist in mitigating future climate impacts [8].

## 4 Health Infrastructure and Energy Resilience

A stable energy supply is crucial for sustaining life‐saving equipment and ensuring appropriate environmental conditions within healthcare facilities. Climate‐induced energy disruptions, such as increased demand during heatwaves, may pose risks to healthcare operations [6]. Although emergency backup systems can offer temporary relief, they do not address the root causes of energy vulnerability. Measures such as investing in renewable energy sources, passive cooling solutions and climate‐adaptive healthcare facility designs may contribute to enhancing resilience while also reducing the environmental footprint of healthcare operations [4, 5, 9]. However, this dependence on centralised and often carbon‐intensive energy grids introduces a paradox, where adaptation strategies risk reinforcing existing vulnerabilities. Emerging decentralised and hybrid energy models, such as microgrids and solar‐battery systems in health facilities, illustrate how distributed generation can enhance both energy and climate resilience.

## 5 Gaps in Infrastructure Planning and Policy Responses

Despite the growing risk associated with climate change, healthcare infrastructure planning does not always fully account for key threats, including heatwaves, energy disruptions and cascading failures [2, 6]. Policy frameworks often focus on hazard‐specific resilience, potentially overlooking the interconnected, cascading and compounding nature of climate risks [2, 6]. A more comprehensive, cross‐sectoral approach that integrates healthcare resilience into climate resilience, the sustainable development goals (e.g., SDG3 Good Health and Well‐Being, SDG13 Climate Action) [10] and disaster risk reduction strategies (i.e., Sendai Framework) [11] would be beneficial. Strengthening healthcare infrastructure to better withstand climate risks could play an important role in protecting public health.

## 6 Strategies for Strengthening Healthcare Resilience

Institutional, political and financial barriers, including fragmented governance, short funding cycles and limited technical capacity, continue to hinder effective coordination across sectors. Lessons from the Sendai Framework [11], COP28 UAE Declaration on Climate and Health [12] and the Alliance for Transformative Action on Climate and Health (ATACH) [13] illustrate the need for alignment between health system resilience and broader climate adaptation governance. Addressing these challenges may require coordinated efforts across multiple sectors, integrating health resilience within broader infrastructure and sustainability planning.

A strategic approach informed by risk assessments and sustainability considerations could help ensure that healthcare facilities remain functional during disasters. Embedding resilience within policy frameworks through a ‘Health in All Policies’ approach may also facilitate integration across different sectors, strengthening national, regional and local adaptation strategies [14].

Recognising the interdependencies between healthcare infrastructure, climate adaptation and sustainable development is crucial for achieving long‐term resilience [15]. Aligning infrastructure investment with academic research, sustainability goals and climate adaptation measures is needed to strengthen healthcare systems [16, 17]. Without further action, critical healthcare infrastructure will remain vulnerable, compromising its ability to provide essential services during disasters. Integrating resilience considerations into healthcare infrastructure planning is therefore critical for supporting system sustainability and stability in an increasingly uncertain future [17].

Table 1 outlines examples of core strategies to support healthcare infrastructure resilience, providing descriptions and examples of potential implementation approaches. Feasibility and cost considerations are noted, acknowledging disparities between high‐income and low‐ and middle‐income settings. Acknowledging the structural inequalities that shape global adaptation capacity, the table's recommendations should be interpreted with recognition that many strategies require targeted international support for low‐ and middle‐income countries to develop climate‐resilient infrastructure. It also evaluates each strategy's feasibility, maturity and scalability across diverse contexts, drawing on empirical insights from recent climate‐related disasters.

**TABLE 1:** Examples of core strategies for strengthening critical health infrastructure.

Strategy type	Strategy	Description	Contribution to resilience	Example	Feasibility/Cost
Foundational	Climate‐adaptive design	Integrate climate resilience measures such as flood‐resistant construction, passive cooling systems, and improved ventilation	Physical robustness against climate hazards	Flood barriers; green roofs; elevated healthcare facilities	Moderate/High
Foundational	Sustainable energy integration	Transition to renewable energy sources and implement energy‐efficient designs	Onsite power continuity and reduced exposure	Solar panels on roofs; wind turbines	Moderate
Enabling	Emergency preparedness and response	Develop emergency preparedness and response plans and maintain stocks of essential supplies	Rapid recovery and adaptive capacity	Staff training and drills for extreme weather events; local warehouses for medical stockpiles	Low/Moderate
Enabling	Cross‐sectoral collaboration	Strengthen cooperation between health, infrastructure, environmental and urban sectors	Systems integration and governance coherence	Climate–health task forces (urban planners, public health experts, climate scientists)	Moderate
Context‐specific	Technological innovation	Invest in smart infrastructure, telemedicine and data‐driven decision making	Anticipatory action and real‐time adaptation	Telehealth services; artificial intelligence (AI)–climate risk health alerts^a^	Moderate/High
Context‐specific	Equity and access	Improve healthcare services in rural and underserved areas	Resilience of care delivery in marginalised areas	Mobile health clinics; telemedicine solutions	Variable
Enabling	Governance frameworks	Create regulations requiring climate resilience in healthcare planning	Policy mandates for resilient infrastructure	Climate‐adaptive building codes	Moderate
Enabling	Training and engagement	Provide climate risk training for healthcare professionals and engage patients and communities	Local adaptation through capacity‐building	Disaster preparedness workshops; climate risk training programmes	Low

^a^ AI growth increases electricity demand and thus system risk.

## 7 Conclusion

Priority investment areas include energy‐secure critical care units, climate‐adaptive facility design and resilient supply chain networks. Preparing healthcare systems to withstand current and future climate challenges requires a paradigm shift in both planning and governance, such as targeted investments and clear policy mandates. Building resilient healthcare infrastructure plays a significant role in disaster response and long‐term public health protection. Efforts to integrate climate resilience strategies, invest in sustainable technologies, and foster cross‐sectoral collaborations are key to strengthening healthcare resilience. These measures enhance the system's ability to adjust, reorganise and retain core functions (i.e., systemic flexibility). In the absence of such proactive measures, climate change will continue to pose increasing challenges, potentially leaving healthcare systems more vulnerable and putting populations at greater risk. Future research should assess the effectiveness and cost–benefit trade‐offs of implemented strategies between sustaining short‐term operational continuity and stability, and enabling longer‐term systemic flexibility and transformation while also developing integrated resilience metrics and appropriate financing mechanisms for low‐resource contexts.

## Author Contributions

**Ana Raquel Nunes:** Conceptualisation; methodology; formal analysis; writing – original draft; writing – review and editing.

## Funding

The author has nothing to report.

## Ethics Statement

The author has nothing to report.

## Conflicts of Interest

The author declares no conflicts of interest.

## Data Availability

Data sharing is not applicable to this article, as no datasets were generated or analysed during the current study.
